# Reference‐Group Adjusted Behavioural Dysfunction Questionnaire Score Discriminates Highly Behavioural‐Variant Frontotemporal Dementia From Major Depressive Disorder and Alzheimer's Disease Dementia

**DOI:** 10.1111/ene.70424

**Published:** 2025-11-10

**Authors:** Anna Semenkova, Olivier Piguet, Andreas Johnen, Matthias L. Schroeter, Jannis Godulla, Christoph Linnemann, Markus Baumgartner, Markus Otto, Ansgar Felbecker, Steven Wezel, Reto W. Kressig, Manfred Berres, Marc Sollberger

**Affiliations:** ^1^ Memory Clinic, University Department of Geriatric Medicine FELIX PLATTER Basel Switzerland; ^2^ Faculty of Psychology, University of Basel Basel Switzerland; ^3^ The University of Sydney, School of Psychology and Brain and Mind Centre Sydney New South Wales Australia; ^4^ Clinic for Neurology, Münster University Hospital Münster Germany; ^5^ Clinic for Cognitive Neurology, University Hospital Leipzig Leipzig Germany; ^6^ Max Planck Institute for Human Cognitive and Brain Sciences Leipzig Germany; ^7^ University Psychiatric Clinic Basel Switzerland; ^8^ Memory Clinic Sonnweid, Sonnweid AG Wetzikon Switzerland; ^9^ Department of Neurology University of Ulm Ulm Germany; ^10^ Department of Neurology University Hospital Halle Halle Germany; ^11^ Clinic of Neurology Und Neurophysiology, Canton Hospital St. Gallen Gallen Switzerland; ^12^ Faculty of Mathematics and Technology, University of Applied Sciences Koblenz Koblenz Germany; ^13^ Department of Neurology University Hospital Basel Basel Switzerland

**Keywords:** Alzheimer dementia, early diagnosis, frontotemporal dementia, major depressive disorder, surveys and questionnaires

## Abstract

**Background:**

Given the fact that behavioural variant frontotemporal dementia (bvFTD) is characterised by behavioural disorders, the assessment of these disorders is essential for early diagnosis of bvFTD. In this regard, the recently developed Behavioural Dysfunction Questionnaire (BDQ) that captures the bvFTD‐specific behavioural disorders is promising in discriminating mild‐stage bvFTD from other neurodegenerative and psychiatric disorders. In this study, we aimed to increase the discriminatory power of the BDQ by adaptation of its scoring depending on the reference group to bvFTD.

**Methods:**

In this combined prospective and retrospective cross‐sectional study, data of 241 patients [i.e., 50 patients with mild‐stage bvFTD, 71 patients with major depressive disorder (MDD) and 120 patients with mild‐stage Alzheimer's disease dementia (ADD)] were analysed. We calculated the BDQ score in two ways: (1) as the average score of the domains' mean scores and (2) by adjusting the scoring depending on the reference group by using machine learning techniques, validated by fivefold cross‐validation.

**Results:**

The adjusted BDQ score showed a higher (bvFTD vs. MDD) or similar (bvFTD vs. ADD) discriminatory power than the unadjusted BDQ score, with a considerably smaller difference between cut‐offs with at least 90% sensitivity and at least 90% specificity.

**Conclusions:**

We recommend using adjusted BDQ scores when MDD or ADD are the reference groups to bvFTD. Similar approaches should be taken for other reference groups to bvFTD to best reflect the thinking of clinicians who have specific reference groups in mind as differential diagnoses to bvFTD.

## 1 Introduction

Behavioural variant frontotemporal dementia (bvFTD) is one of the most common younger‐onset dementias, with the first symptoms occurring before the age of 65 [1]. It is characterised by prominent changes in personality and behaviour, with often relatively intact up to unspecific performance on ‘classical’ neuropsychological tests [2]. Due to this and the lack of bvFTD‐specific biomarkers for sporadic (i.e., nongenetic) cases, patients receive the correct diagnosis often with up to 6 years delay after symptom onset [3]. Misdiagnosis is common, with about 50% of bvFTD patients initially receiving a psychiatric diagnosis, most of all, major depressive disorder (MDD) [4]. Conversely, around 50% of patients with other neurodegenerative disorders, such as atypical Alzheimer's disease dementia (ADD) or psychiatric disorders such as MDD, are first misdiagnosed with bvFTD [5, 6].

To increase the sensitivity to bvFTD symptoms, the International Behavioural Variant FTD Criteria Consortium (FTDC) developed revised diagnostic criteria in 2011, including five behavioural domains and one cognitive domain [7]. However, without tools that operationalise the assessment of the behavioural domains, the application of these criteria remains challenging, especially for people with little expertise in bvFTD.

To overcome this challenge, we have recently developed the Behavioural Dysfunction Questionnaire (BDQ) [8], an informant‐based questionnaire that operationalises the five FTDC behavioural diagnostic criteria [7] using a Likert scale. The BDQ was found to differentiate well between mild‐stage bvFTD patients and mild‐stage ADD patients as well as MDD patients, respectively [8]. However, the sensitivity and specificity of the cut‐off scores provided by the Youden index were lower than expected (bvFTD vs. MDD: sensitivity = 76%, specificity = 78%; bvFTD vs. ADD: sensitivity = 79%; specificity = 88%) [8], implying shared behavioural features between bvFTD and the two other diagnostic groups. Indeed, the primary aim of the revised FTDC diagnostic criteria was to increase the sensitivity to bvFTD, whereas the specificity was less in focus [7, 9]. One study that applied the revised diagnostic criteria for possible bvFTD (relying solely on clinical domains) in postmortem confirmed cases showed a specificity of 82% at a sensitivity of 95% [10]. However, in another study that also included psychiatric disorders, the specificity was only 27% at a sensitivity of 85% [11]. Notably, in these studies, as in our previous study [8], all clinical characteristics for bvFTD had the same diagnostic weight. However, depending on the reference group with which the bvFTD patients are compared, behavioural features from the revised FTDC diagnostic criteria overlap more (e.g., altered eating behaviours are core symptoms in mild‐stage bvFTD and MDD [7, 12]) or less (e.g., eating behaviours are typically not altered in mild‐stage ADD [13, 14]). Therefore, adjustment of the BDQ scoring depending on the reference group to bvFTD should increase the specificity of the BDQ and, therefore, improve its discriminatory power.

In this study, we took this approach by adjusting the BDQ‐scoring depending on the reference group to bvFTD (i.e., MDD, ADD) by means of machine learning techniques. In a second step, we compared the discriminatory power of the adjusted BDQ score to the unadjusted BDQ score, the type of scoring we used in our previous study [8]. And third, we compared the discriminatory power of the adjusted BDQ score with that of two other clinical tools, that is, the Frontal Behavioural Inventory (FBI) [15], the most established behavioural scale used in bvFTD diagnosis and the Montreal Cognitive Assessment (MoCA) [16], a widely used cognitive screening test.

## 2 Methods

### 2.1 Participants

To pursue a data‐driven scoring approach with five‐fold cross‐validation, while recognising the rarity of bvFTD cases [1], we set the required number of bvFTD patients at 50 (i.e., 10 patients per cross‐validation round). Within the time period required to recruit the bvFTD patients, we recruited as many ADD (≤ 80 years of age) and MDD patients (≥ 45 years of age) as possible. Patients were enrolled within our previous study (51%) [8] and later within clinical routine (49%).

By taking this approach, we included 241 patients of European origin from several Swiss and German medical centres with expertise in early diagnosis of bvFTD, ADD and/or MDD in the analysis. With the exception of one psychiatric centre, where 18 MDD patients (25% of MDD patients and 7% of all patients) were recruited, all recruitment sites assessed patients with cognitive disorders of various origins, including those associated with psychiatric and neurodegenerative diseases.

The sample consisted of 50 bvFTD patients (47 probable bvFTD and 3 bvFTD with definite frontotemporal lobar degeneration pathology [7]) with a major neurocognitive disorder at mild stage according to DSM‐5 [12], 71 patients with MDD (40 moderate depressive episode and 31 severe depressive episode according to ICD‐10 [17]) and 120 typically amnestic ADD patients (15 probable ADD, 104 probable ADD with evidence of AD pathophysiology and one pathophysiologically proved ADD [14]) with a major neurocognitive disorder at mild stage according to DSM‐5 [12]. Ten (8.5%) of the 120 ADD patients fulfilled the 2021 International Working Group recommendations for AD [18]. According to the Amyloid‐Tau‐Neurodegeneration (ATN) system [19], ADD patients were subclassified as follows: 8.5% as A+T+N+, 2.5% as A+T+N‐, 6.5% as A+T‐N+, 5% as A+T‐N‐, 3.5% as A‐T+N+, 72.5% as A‐T‐N+ and 1.5% as A‐T‐N‐.

Additional inclusion criteria for all patients were the availability of a reliable informant (> 18 years) who has regular contact with the patient. Specific exclusion criteria for bvFTD and ADD patients were a major neurocognitive disorder at or above moderate stage according to DSM‐5, history of severe depressive episode or current depressive episode according to ICD‐10, and history of or current major psychiatric disorders according to ICD‐10. Specific exclusion criteria for MDD patients were a neurocognitive disorder according to DSM‐5 and any other major psychiatric disorders according to ICD‐10. Exclusion criteria for all patient groups were a history of or current drug and/or alcohol abuse as well as drug‐ or alcohol‐related disorders according to ICD‐10 and traumatic brain injuries, systemic disorders or brain diseases that could result in behavioural changes. Patients' inclusion and exclusion criteria are listed in (Supporting Information A).

On average, the time from the first symptoms to data assessment was 41.07 (±27.59) months for bvFTD patients, 30.86 (±23.98) months for ADD patients and 18.11 (±21.34) months for MDD patients.

Patients were assessed by an interdisciplinary team comprising either neurologists, psychiatrists and neuropsychologists, or neurologists and neuropsychologists, except at the one psychiatric centre focusing on psychiatric disorders. At this centre, patients were primarily assessed by psychiatrists, with interdisciplinary assessments by psychiatrists and neuropsychologists being rare.

All bvFTD and ADD patients, as well as 55% of the MDD patients, underwent a comprehensive neuropsychological examination that included the cognitive domains of psychomotor speed, attention (simple and divided), memory (verbal‐episodic and visual‐episodic), language, executive functions, as well as visuo‐perceptual and visuo‐constructive functions. The general cognitive ability of all patients was assessed using either the MMSE (53%) [20] or the MoCA (47%) [16]. Mood and behaviour were assessed through clinical observation and self‐ and informant‐report questionnaires. Please see the neuropsychological tests with the percentages of patients who were tested with each test in (Supporting Information B).

The standard neuroimaging assessment was a MRI scan. Of all patients, 24 MDD patients (33% of MDD patients) did not undergo brain MRI scanning. Of these, two patients underwent CT scanning, leaving 22 MDD patients (31% of MDD patients and 9% of all patients) without brain imaging. No brain imaging was performed on these patients as they were known to have recurrent depressive disorder. Notably, 17 of these 22 patients (77%) underwent diagnostic re‐evaluation to confirm the diagnosis. Further diagnostic investigations were performed depending on the clinical and structural imaging findings. These included neuroimaging (e.g., FDG‐PET, amyloid‐PET and/or tau‐PET), cerebrospinal fluid (including amyloid‐*β*1‐42, the amyloid‐*β*1‐42/1–40 ratio, p‐tau181 and/or tau) and serum neurofilament light chain analyses.

To increase diagnostic certainty, we confirmed 77% of patients' diagnoses [72% of bvFTD (mean time period of 2.29 ± 1.10 years), 76% of ADD (mean time period of 2.15 ± 0.87 years), 82% of MDD (mean time period of 2.42 ± 0.97 years)] by a follow‐up assessment either in institutions or by a standardised phone interview (mean time period of 2.26 ± 1.00 years).

The study was approved by the Ethics Committee of Northwestern and Central Switzerland (EKNZ). Subjects and their close ones gave informed consent for participation.

### 2.2 Behavioural and Cognitive Tools

#### 2.2.1 Behavioural Dysfunction Questionnaire (BDQ)

BDQ [8] is an informant‐report questionnaire based on the five behavioural domains of the FTDC diagnostic criteria for bvFTD [7]. Items of each behavioural domain are scored for their frequency or severity (depending on what is more appropriate for grading the behavioural disorder) on a Likert scale from 0 (none) to 5 (very often/very severe). The total score is the average score of the five domains' mean scores. The questionnaire is presented in (Supporting Information C).

#### 2.2.2 Frontal Behavioural Inventory (FBI)

The FBI [15] is one of the most established behavioural scales used for the clinical evaluation of cases suspected of bvFTD. The FBI contains 24 items scored on a Likert scale from 0 (none) to 3 (severe/most of the time) and the total score is the sum of all items. Here, the informant‐report questionnaire version of the FBI was used.

#### 2.2.3 Montreal Cognitive Assessment (MoCA)

MoCA [16] is a cognitive screening test often used in the clinical evaluation of patients with suspected neurocognitive disorders. It briefly evaluates different cognitive skills, such as memory, visuospatial functions, verbal fluency, etc. The total score is the sum of all cognitive tasks.

Of note, 54% of the patients (35% of bvFTD, 53% of MDD and 62% of ADD) were not assessed with the MoCA, but with the Mini Mental Status Examination (MMSE) [20]. MMSE scores of these patients were converted into MoCA scores [21].

### 2.3 Statistical Approach

The first set of analyses focused on the comparison between bvFTD and MDD, the second set on the comparison between bvFTD and ADD.

### 2.4 Discriminatory Power of the BDQ Without Scoring Adjustment

According to Semenkova et al. [8], we calculated the BDQ score as the average score of the five domains' mean scores. Following this, we performed univariate logistic regressions with the BDQ score as a predictor variable, followed by ROC curve analyses. We then compared the discriminatory power of the unadjusted BDQ scores obtained in this study with those of the previous study [8] to test the reliability of our previous analyses with the unadjusted BDQ score.

### 2.5 BDQ Score Adjustment According to the Reference Group

We performed the adjustments of the BDQ score depending on the reference group (MDD or ADD) to bvFTD in five steps.

#### 2.5.1 Step 1

We analysed the discriminatory power of each item between bvFTD and the reference group, applying Kendall‐Tau rank correlation coefficients [22]. Items with *p* > 0.2 were excluded as assumed to have too low discriminatory power between the diagnostic groups [23].

#### 2.5.2 Step 2

Using the retained items, we generated subdomains' mean scores and performed univariate logistic regressions with each subdomain's score separately. If the 95% confidence interval of the regression coefficient included 0, the subdomain was excluded from further analysis.

#### 2.5.3 Step 3

Using the retained subdomains, we calculated domains' mean scores and performed lasso (least absolute shrinkage and selection operator) regression analyses [24] with the domains' scores as predictor variables. The lasso method allows applying a penalty term to predictive variables to avoid overoptimistic regression coefficients, resulting in a shrinkage of these coefficients. If a coefficient is shrunken to 0, the variable is excluded from the model. This method makes the results more generalisable.

#### 2.5.4 Step 4

Next, we weighted the domains in the predictive model by multiplying the domains' mean scores with regression coefficients from the previously performed lasso analysis. By that, the domains' scores with higher predictive power received more weight in the final score than the scores with lower predictive power.

#### 2.5.5 Step 5

We calculated the BDQ score as the average score of the weighted domains' mean scores generated in step 4 and ran a univariate logistic regression followed by ROC curve analysis with the BDQ score as a predictor.

### 2.6 Validation of the Applied Scoring Approach

We performed fivefold cross‐validation to validate the scoring method described above and to ensure the generalisability of the results. We used 80% of the sample to train the model (Steps 2 and 3) and 20% of the sample to test the model (Steps 4 and 5) using the largest value of lambda such that the mean squared error is within 1 standard error of the minimal mean squared error (Step 3). This procedure was repeated five times with disjoint test samples. Mean AUC as well as mean sensitivity and mean specificity (calculated with the Youden index) of the five test models were compared with those of the respective subsamples.

### 2.7 Comparison of the Discriminatory Power of the Adjusted BDQ, FBI and MoCA

As data from all three tools were not available for all patients, the comparisons of the discriminatory power of the adjusted BDQ, FBI and MoCA were carried out in a subsample of 214 patients (*n*_bvFTD_ = 40, *n*_MDD_ = 62, *n*_ADD_ = 112). Three univariate logistic regression analyses with each score separately, followed by ROC curve analyses, were performed. The results of the three ROC curve analyses were compared using DeLong's method [25].

## 3 Results

Analysis of covariance followed by Tukey–Kramer post hoc analysis for education and Chi‐square test for sex showed no differences among patient groups (Table 1). ADD patients were on average older than bvFTD and MDD patients, as shown by Kruskal–Wallis test followed by the pairwise Wilcoxon test. BvFTD and ADD patients were cognitively more impaired than MDD patients, and ADD patients were cognitively more impaired than bvFTD patients, as measured by MoCA [16]. BvFTD patients had higher FBI scores [15] than ADD and MDD patients (Table 1).

**TABLE 1:** Demographic and clinical characteristics of study participants (*N* = 241) classified by diagnostic group.

Diagnosis	Mild‐stage bvFTD (*n* = 50)	MDD (*n* = 71)	Mild‐stage ADD (*n* = 120)	Statistic test (df)	Post Hoc
Age (years)	64.02 ± 10.15	64.2 ± 9.77	70.46 ± 9.32	30.2 _(2)_ ^a^, **	ADD >bvFTD, MDD**
Sex (m/f)	27/23	34/37	48/72	3.08 _(2)_ ^b^	
Education (years)	13.77 ± 2.76	13.33 ± 3.26	12.82 ± 3.39	1.6 _(2,228)_ ^c^	
MoCA (0–30)	19.13 ± 5.88	24.27 ± 4.24	16.81 ± 5.05	44.52 _(2,220)_ ^c^, **	MDD > bvFTD, ADD** bvFTD > ADD*
FBI (0–72)	26.35 ± 12.01	11.39 ± 8.77	11.8 ± 7.56	50.82 _(2)_ ^a^, **	bvFTD > ADD, MDD**

*Note:* All bvFTD and ADD patients were diagnosed with major neurocognitive disorder at mild stage according to DSM‐5.

Abbreviations: ADD, Alzheimer's disease dementia; bvFTD, behavioural variant frontotemporal dementia; FBI, Frontal Behavioural Inventory; MDD, major depressive disorder; MoCA, Montreal Cognitive Assessment.

^a^ Kruskal–Wallis test.

^b^ Chi‐square test.

^c^ Analysis of variance.

* *p* < 0.05.

** *p* < 0.001.

### 3.1 Discriminatory Power of the Adjusted BDQ Score

#### 3.1.1 bvFTD Versus MDD

**FIGURE 1:**
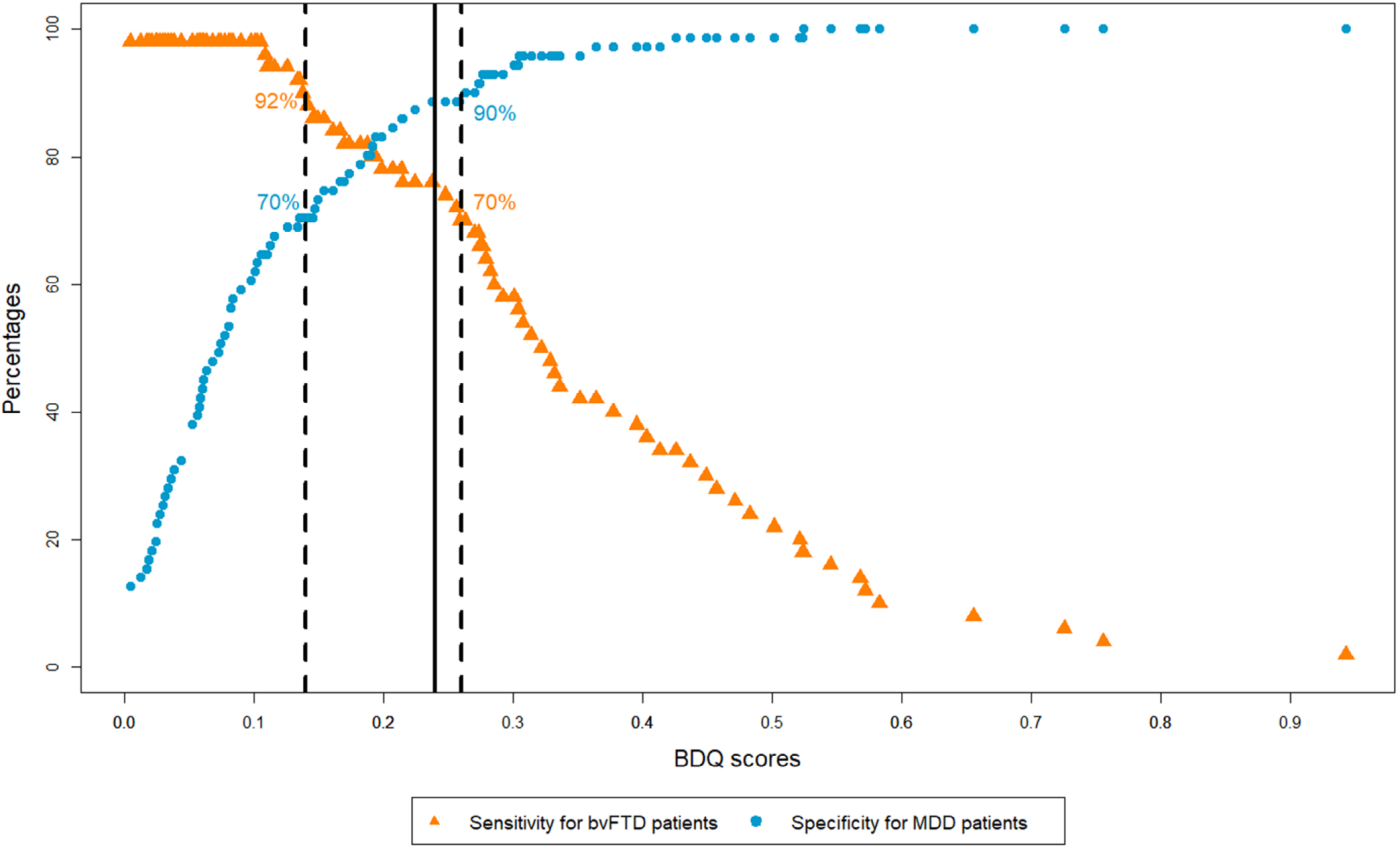
Cut‐offs between bvFTD and MDD patients.

After Kendall‐Tau analyses, five items (i.e., two items from the domain ‘disinhibition’ and three items from the domain ‘stereotypical, perseverative behaviour’) were excluded from further scoring (see Supporting Information D for more details). Separate univariate logistic regressions with each subdomain's mean score showed that all subdomains discriminated between bvFTD and MDD (Supporting Information E). The lasso regression analysis resulted in the exclusion of domain C (loss of empathy) (Table 2). Among the remaining domains, domain A (disinhibition) showed the highest discriminatory power with a regression coefficient of 0.82. The adjusted BDQ score discriminated better between bvFTD and MDD patients than the unadjusted score based on Delong's method (AUC = 89.56 vs. AUC = 83.76, difference of 5.8 with 95% confidence interval (CI) [1.57, 10.04]), associated with a considerably smaller difference between cut‐off scores with at least 90% sensitivity and at least 90% specificity (Table 3, Figure 1).

**TABLE 2:** Lasso regression analyses with domains' mean scores.

	Regression coefficients	
	bvFTD vs. MDD	bvFTD vs. ADD
Domain A	0.82	0.79
Domain B	0.13	0.42
Domain C	.	0.07
Domain D	0.24	0.27
Domain E	0.08	0.24

Abbreviations: ADD, Alzheimer's disease dementia; bvFTD, behavioural variant frontotemporal dementia; Domain A, Early behavioural disinhibition; Domain B, Early apathy or inertia; Domain C, Early loss of sympathy or empathy; Domain D, Early perseverative, stereotyped or compulsive/ritualistic behaviour; Domain E, Hyperorality and dietary changes; MDD, major depressive disorder.

**TABLE 3:** Discriminatory power of the BDQ score in Semenkova et al. [8] and in the present subsamples calculated according to Semenkova et al. [8] (unadjusted score) and adjusted to the reference group using machine learning techniques.

	bvFTD vs. MDD			bvFTD vs. ADD		
	Semenkova et al. (*n*_bvFTD_ = 34, *n*_MDD_ = 41)	Present sample (*n*_bvFTD_ = 50, *n*_MDD_ = 71)		Semenkova et al. (*n*_bvFTD_ = 34, *n*_ADD_ = 56)	Present sample (*n*_bvFTD_ = 50, *n*_ADD_ = 120)	
		Unadjusted score	Adjusted score		Unadjusted score	Adjusted score
Area under the curve, %	83.43 (CI: 74.37–92.49)	83.76 (CI: 76.72–90.8)	89.56 (CI: 83.75–95.38)	87.84 (CI: 80.48–95.2)	91.22 (CI: 86.3–96.13)	92.13 (CI: 87.32–96.95)
Cut‐off score by Youden Index (SE/SP)	1.1 (76%/78%)	1.1 (78%/75%)	0.24 (76%/89%)	1 (79%/88%)	1 (80%/89%)	0.27 (90%/83%)
Cut‐off score with at least 90% sensitivity (SE/SP)	0.6 (91%/56%)	0.6 (92%/59%)	0.14 (92%/70%)	0.6 (91%/59%)	0.6 (90%/73%)	0.27 (90%/83%)
Cut‐off score with at least 90% specificity (SE/SP)	1.6 (56%/90%)	1.6 (56%/90%)	0.26 (70%/90%)	1.4 (65%/91%)	1.1 (76%/92%)	0.36 (78%/91%)

Abbreviations: ADD, Alzheimer's disease dementia; bvFTD, behavioural variant frontotemporal dementia; CI, 95% confidence interval. MDD, major depressive disorder; SE; sensitivity; SP, specificity.

We found a score of > 0.26 to be strongly indicative of bvFTD (sensitivity 70%, specificity 90%) and a score of < 0.14 to be strongly indicative of MDD (sensitivity 92%, specificity 70%). Scores between 0.14 and 0.26 were considered equivocal (Figure 1).

#### 3.1.2 bvFTD Versus ADD

After Kendall‐Tau analyses, five items (i.e., the same two items from the domain ‘disinhibition’ as in the bvFTD vs. MDD analyses and three items from the domain 'stereotypical, perseverative behaviour' of which two were the same as in the bvFTD vs. MDD analyses) were excluded from further scoring (see Supporting Information D).

**FIGURE 2:**
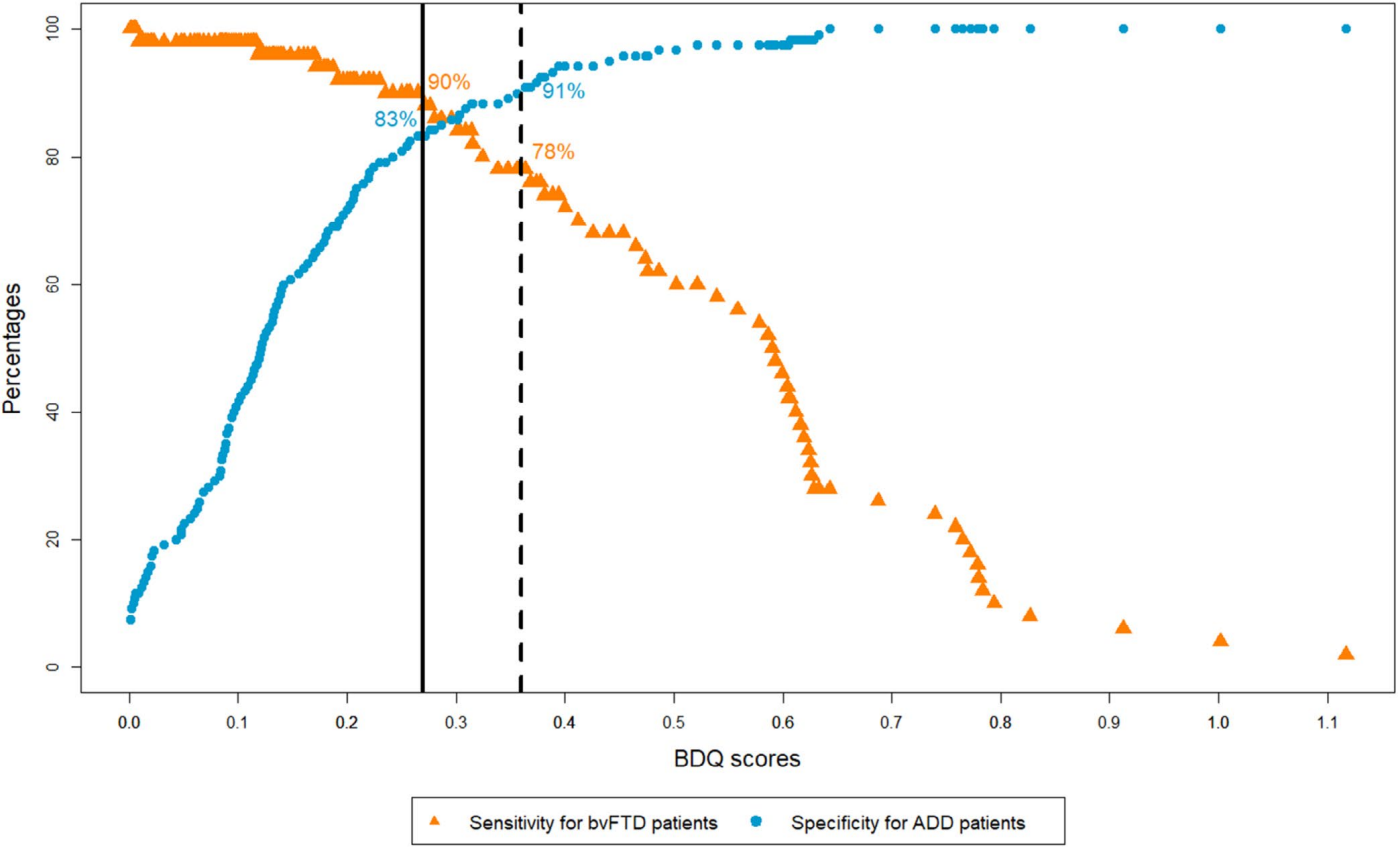
Cut‐offs between bvFTD and ADD patients.

Separate univariate logistic regressions with each subdomain's mean score involving the retained items showed that all subdomains significantly discriminated between bvFTD and ADD (Supporting Information E). Similarly, lasso regression analysis kept all domains in the model, thereby empowering domain A (disinhibition) the most (regression coefficient 0.79) and empowering domain C (loss of empathy) the least (regression coefficient 0.07) (Table 2). The discriminatory power of the adjusted BDQ score was similar to the one without adjustment, but the difference between cut‐off scores with at least 90% sensitivity and at least 90% specificity was smaller using the adjusted BDQ score (Table 3, Figure 2).

We found a score of > 0.36 to be strongly indicative for bvFTD (sensitivity 78%, specificity 91%) and a score of < 0.27 to be strongly indicative for ADD (sensitivity 90%, specificity 83%). Scores between 0.27 and 0.36 were considered equivocal (Figure 2).

### 3.2 Validation of the Applied Scoring Approach Using Fivefold Cross‐Validation

#### 3.2.1 bvFTD Versus MDD

The average AUC for the five test models was 86.55% which was slightly smaller than the one in the subsample (89.56%). When examining the results of each test round, we found similar AUC scores to the one in the subsample in four test rounds, but a lower AUC score (76.79%) in one test round (Supporting Information F). Of the five test models together, the average sensitivity was 90% and the average specificity was 85% for the cut‐off scores calculated with the Youden index, compared to 76%–89% in the subsample respectively.

The small differences in reported values between cross‐validation and the subsample are likely explained by the small sample sizes of the five test models ( *n*_bvFTD_ = 10, *n*_MDD_ = 14) in the cross‐validation which increases the risk of potential outliers.

#### 3.2.2 bvFTD Versus ADD

The average AUC of the five test models was 91.34% (compared to 92.13% in the subsample) with an average sensitivity of 90% and an average specificity of 88% for the cut‐off scores calculated by the Youden index, compared to 90% and 83% in the subsample respectively (Supporting Information G).

### 3.3 Comparing the Discriminatory Power of the Adjusted BDQ, FBI and MoCA

#### 3.3.1 bvFTD Versus MDD

**FIGURE 3:**
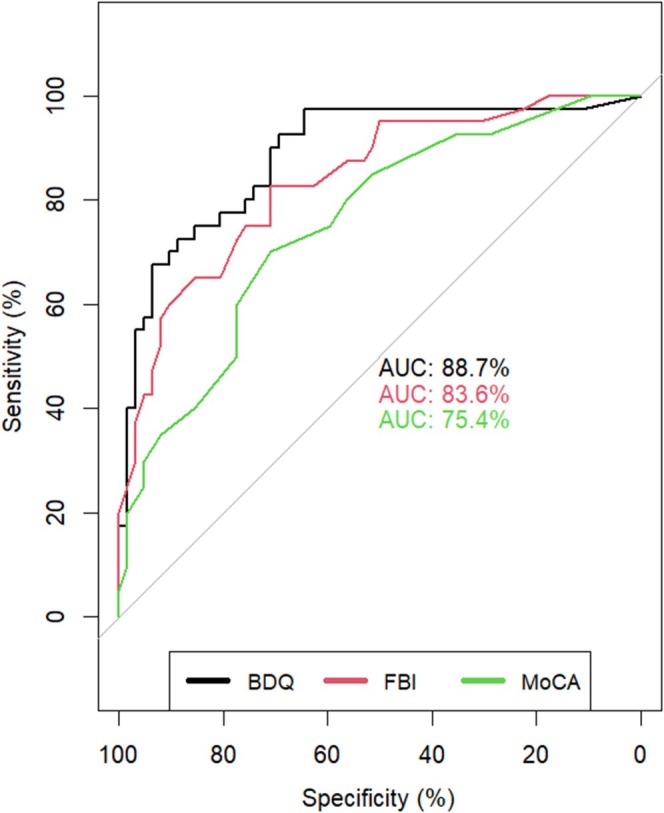
Comparison of the area under curve of the three diagnostic instruments between bvFTD and MDD patients.

Using Delong's method for comparison of the AUC scores, the adjusted BDQ score outperformed the scores of the FBI and the MoCA, that is, the AUC for the adjusted BDQ score was 88.7% compared to that for the FBI score at 83.6% (difference of 5.1, 95% CI [0.7, 9.4]) and to that for the MoCA score at 75.4% (difference of 13.3, 95% CI [2.2, 24.4]) (Figure 3). The discriminatory powers of the FBI and MoCA were less distinct (difference of 8.2, 95% CI [−3.4, 19.9]).

#### 3.3.2 bvFTD Versus ADD

**FIGURE 4:**
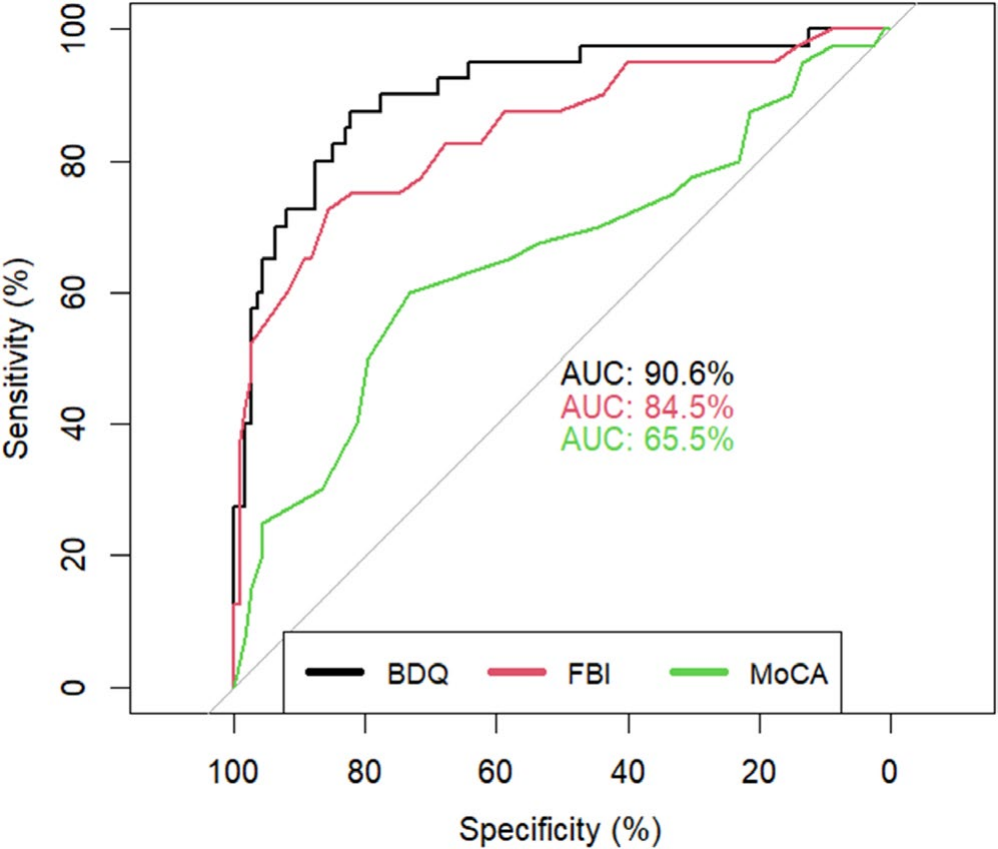
Comparison of the area under curve of the three diagnostic instruments between bvFTD and ADD patients.

As in the bvFTD‐MDD subsample, the adjusted BDQ score outperformed the scores of the two other tools, that is, the AUC for the adjusted BDQ score was 90.6% compared to that for the FBI score with 84.5% (difference of 6.1, 95% CI [1.5, 10.5]) and to that for the MoCA score with 65.5% (difference of 25.1, 95% CI [12.6, 37.6]) (Figure 4). In addition, the FBI discriminated bvFTD from ADD patients better than the MoCA (difference of 19.0, 95% CI [5.3, 32.9]).

## 4 Discussion

By using a reference‐adjusted scoring approach, we increased the power of the BDQ to differentiate mild‐stage bvFTD from MDD and mild‐stage ADD, respectively, in comparison to the unadjusted BDQ score. The adjusted BDQ score, which was validated by fivefold cross‐validation, demonstrated excellent [26] discriminatory power between mild‐stage bvFTD and MDD patients (AUC = 90%) and between mild‐stage bvFTD and mild‐stage ADD patients (AUC = 92%) respectively.

In addition, we showed that the discriminatory power of the unadjusted BDQ scores in the subsamples was similar to the discriminatory power of the ones reported in our previous study [8], supporting the reliability of the results of our previous study using the BDQ.

Lastly, our analyses determined that the adjusted BDQ scoring discriminated better between the groups than the widely used FBI [15], an informant‐report questionnaire on behavioural disorders in bvFTD and the MoCA [16], a widely used cognitive screening test. These findings support the use of the BDQ and its adjusted scoring method over the FBI and the MoCA when aiming to differentiate mild‐stage bvFTD from MDD and mild‐stage ADD.

Another clinical tool, which refers to the revised FTDC diagnostic criteria for bvFTD [7], is DAPHNE [27]. DAPHNE, a standardised informant interview, was found to discriminate between bvFTD and ADD patients similarly to the FBI [27]. In our study, however, the FBI discriminated less well than the adjusted BDQ score. This means that DAPHNE would likely discriminate bvFTD from ADD patients less well than the adjusted BDQ score. We cannot compare the discriminatory power of the BDQ and DAPHNE to other reference groups to bvFTD for lack of published data.

Despite the high discriminatory power of the adjusted BDQ score, we need to consider the number of patients who were misclassified by the BDQ, and this in reference groups to bvFTD with very low diagnostic uncertainty. These findings suggest that the five behavioural disorders of the revised FTDC diagnostic criteria [7], as assessed by the BDQ, are of limited specificity. Combining the BDQ with other clinical tools that encompass clinical features, listed as supportive in the revised FTDC diagnostic criteria, such as ‘loss of (socio‐emotional) insight’ or ‘social cognition’, may help to increase the discriminatory power [7, 9, 28, 29, 30].

Arguably, our study is limited by the absence of postmortem pathological confirmations of our patients' diagnoses. Accordingly, we cannot exclude the possibility of misdiagnosis in our patients, an important issue in bvFTD, where misdiagnosis early in the disease process is not uncommon [5, 6]. To increase diagnostic certainty, we confirmed the diagnoses in 77% of the patients and 72% of the bvFTD patients, respectively, by follow‐up assessments.

In conclusion, we demonstrated that the BDQ score, either adjusted to MDD or to ADD as a reference group to bvFTD, differentiates bvFTD reliably and to a high degree from these two diagnostic groups. Similar analytical approaches, not only for the scoring of the BDQ, but also for other clinical instruments, regarding other reference groups to bvFTD likely to be misdiagnosed with bvFTD, such as bipolar disorder [11], right‐temporal variant frontotemporal dementia [31] or behavioural variant AD should be applied. Scoring clinical instruments in this way not only improves their discriminatory power but also reflects clinicians' thinking about the differential diagnosis of bvFTD.

## Author Contributions

**Anna Semenkova:** conceptualisation; data curation; formal analysis; investigation; methodology; project administration; visualisation; writing, original draft; writing, review and editing. **Olivier Piguet:** validation; writing, review and editing. **Andreas Johnen:** investigation; writing, review and editing. **Matthias L. Schroeter:** investigation; writing, review and editing. **Jannis Godulla:** investigation; writing, review and editing. **Christoph Linnemann:** investigation; writing, review and editing. **Markus Baumgartner:** investigation; writing, review and editing. **Markus Otto:** investigation; writing, review and editing. **Ansgar Felbecker:** investigation; writing, review and editing. **Steven Wezel:** formal analysis; writing – review and editing. **Reto W. Kressig:** writing, review and editing. **Manfred Berres:** conceptualisation; formal analysis; methodology; writing, review and editing; validation. **Marc Sollberger:** conceptualisation; data curation; investigation; project administration; resources; supervision; writing, original draft; writing, review and editing; funding acquisition.

## Ethics Statement

The study was approved by the Ethics Committee of Northwestern and Central Switzerland and has been performed in accordance with the ethical standards of the 1964 Declaration of Helsinki and its later amendments. Participants gave informed consent to participate in the study before taking part.

## Conflicts of Interest

The authors declare no conflicts of interest.

## Supporting information


**Data S1:** Supporting Information.

## Data Availability

The data that support the findings of this study are available from the corresponding author upon reasonable request.
