# Supplementary material for: Reference‐Group Adjusted Behavioural Dysfunction Questionnaire Score Discriminates Highly Behavioural‐Variant Frontotemporal Dementia From Major Depressive Disorder and Alzheimer's Disease Dementia
Source: Eur J Neurol. 2025 Nov 10;32(11):e70424. doi: 10.1111/ene.70424 (PMC12598400; doi:10.1111/ene.70424)
Supplement: Supplementary file 1 — Data S1: Supporting Information. [file ENE-32-e70424-s001.docx]

**Supplementary Material to “Reference-group adjusted Behavioural Dysfunction Questionnaire score discriminates highly behavioural-variant frontotemporal dementia from major depressive disorder and Alzheimer's disease dementia”**

Anna Semenkova^1,2^, Olivier Piguet^3^, Andreas Johnen^4^, Matthias L. Schroeter^5,6^, Jannis Godulla^5,6^, Christoph Linnemann^7^, Markus Baumgartner^8^, Markus Otto^9,10^, Ansgar Felbecker^11^, Steven Wezel^1,2^, Reto W. Kressig^1^, Manfred Berres^12^, and Marc Sollberger^1,13^

| *1* | Memory Clinic, University Department of Geriatric Medicine FELIX PLATTER, Basel, Switzerland |
| --- | --- |
| *2* | Faculty of Psychology, University of Basel, Switzerland |
| *3* | The University of Sydney, School of Psychology and Brain and Mind Centre, NSW, Australia |
| *4* | Clinic for Neurology, Münster University Hospital, Münster, Germany |
| *5* | Clinic for Cognitive Neurology, University Hospital Leipzig, Germany |
| *6* | Max Planck Institute for Human Cognitive and Brain Sciences, Leipzig, Germany |
| *7* | University Psychiatric Clinic, Basel, Switzerland |
| *8* | Memory Clinic Sonnweid, Sonnweid AG, Wetzikon, Switzerland |
| *9* | Department of Neurology, University of Ulm, Ulm, Germany |
| *10* | Department of Neurology, University Hospital Halle, Germany |
| *11* | Clinic of Neurology und Neurophysiology, Canton Hospital St. Gallen, Switzerland |
| *12* | Faculty of Mathematics and Technology, University of Applied Sciences Koblenz, Germany |
| *13* | Department of Neurology, University Hospital Basel, Switzerland |

Correspondence concerning this article should be addressed to Marc Sollberger, Memory Clinic, University Department of Geriatric Medicine FELIX PLATTER, Burgfelderstrasse 101, 4055 Basel, Switzerland. Email: marc.sollberger@felixplatter.ch

**table of content**

[Supplementary A. Inclusion and exclusion criteria 4](#_Toc208478399)

[Inclusion criteria for the patients with behavioural variant frontotemporal dementia or Alzheimer’s disease dementia 4](#_Toc208478400)

[Exclusion criteria for the patients with behavioural variant frontotemporal dementia or Alzheimer’s disease dementia 4](#_Toc208478401)

[Inclusion criteria for the patients with major depressive disorder (moderate or severe depressive episode according to ICD-10) 5](#_Toc208478402)

[Exclusion criteria for the patients with major depressive disorder (moderate or severe depressive episode according to ICD-10) 5](#_Toc208478403)

[Supplementary B. Neuropsychological Tests 6](#_Toc208478404)

[Supplementary C. Behavioural Dysfunction Questionnaire 7](#_Toc208478405)

[Supplementary D. Kendall-Tau rank correlation coefficients 13](#_Toc208478406)

[Supplementary E. Univariate logistic regression analyses with subdomains’ mean scores 16](#_Toc208478407)

[Supplementary F. Cross-validation for bvFTD vs. MDD patients 17](#_Toc208478408)

[Supplementary G. Cross-validation for bvFTD vs. ADD patients 19](#_Toc208478409)

# Supplementary A. Inclusion and exclusion criteria

### Inclusion criteria for the patients with behavioural variant frontotemporal dementia or Alzheimer’s disease dementia

- Diagnoses of
  - probable behavioural variant frontotemporal dementia (FTD) or behavioural variant FTD with definite frontotemporal lobar degeneration pathology[1], or
  - probable Alzheimer’s disease dementia (ADD), probable ADD with evidence of AD pathophysiology or pathophysiologically proved ADD[2]
- Major neurocognitive disorder at mild stage according to DSM-5
- Patient is able to understand the informed consent and to judge about using his/her data for research purposes (estimated by examiner)
- Availability of a reliable informant (age ≥ 18), who has regular contact (at least once a week) with the subject

### Exclusion criteria for the patients with behavioural variant frontotemporal dementia or Alzheimer’s disease dementia

- No signed informed consent
- Presence of other conditions that may partly account for the neuropsychological deficits such as:
  - Brain disorders (e.g., stroke, multiple sclerosis, other neurodegenerative diseases than the inclusion diagnosis)
  - History of or current drug/alcohol abuse as outlined in ICD-10
  - History of or current major psychiatric disorders such as bipolar disorder, anxiety disorder, personality disorder, obsessive compulsive disorder, or schizophrenia as outlined in ICD-10
  - Systemic disorders (e.g., heart, pulmonary)
  - Brain trauma
- Mild or more severe depressive episode as outlined in ICD-10
- For ADD patients: ≥ 80 years of age

### Inclusion criteria for the patients with major depressive disorder (moderate or severe depressive episode according to ICD-10)

- Diagnoses of moderate or severe depressive episode according to ICD-10
- Patient is able to understand the informed consent and to judge about using his/her data for research purposes (estimated by examiner)
- Availability of a reliable informant (age ≥ 18), who has regular contact (at least once a week) with the subject

### Exclusion criteria for the patients with major depressive disorder (moderate or severe depressive episode according to ICD-10)

- No signed informed consent
- ≤ 45 years of age
- Presence of other conditions that may partly account for the neuropsychological deficits such as:
  - Brain disorders (e.g., stroke, multiple sclerosis, neurodegenerative diseases)
  - History of or current drug/alcohol abuse as outlined in ICD-10
  - History of or current additional diagnosis of major psychiatric disorders other than the inclusion diagnosis
  - Systemic disorders (e.g. heart, pulmonary)
  - Brain trauma

# Supplementary B. Neuropsychological Tests

Psychomotor speed: Trail Making Test A[3] (85%) , Stroop Part 1[4] (85%)

Alertness: Testbatterie zur Aufmerksamkeitsprüfung[5] (58%)

Simple Attention: WMS-R—verbal digit span, forward[6] (93%), WMS-R—Corsi block, forward[6] (58%)

Divided attention: Testbatterie zur Aufmerksamkeitsprüfung[5] (58%)

Response inhibition: Testbatterie zur Aufmerksamkeitsprüfung[5] (58%)

Memory: CERAD Wordlist[7] (85%), CERAD Constructional Praxis Recall[7] (85%)

Language: Boston Naming Test[6] (85%)

Visuconstruction and visuoperception: CERAD Constructional Praxis[7] (85%)

Executive functions: WMS-R—verbal digit span, backward[6] (85%), WMS-R—Corsi block, backward[6] (60%), Trail Making Test B[3] (85%), TMT B/A Ratio (85%), Stroop Quotient[4] (85%), S-words[8] (85%), Animal fluency[9] (85%), Five points test[10] (85%),

Overall cognitive ability: Mini-Mental State Examination (MMSE)[11] (53%) or Montreal Cognitive Assessment[12] (47%)

Percentages refer to the patients who had performed the respective test

# Supplementary C. Behavioural dysfunction questionnaire

Dear Sir or Madam

With this questionnaire we would like to examine whether **your close person** exhibits any behavioural disorders.

Should a behavioural disorder apply, we would like you to indicate exactly how pronounced that behavioural disorder is.

You may choose from the following answer options:

- Very mild / rare (less than once a month)
- Mild /occasionally (approximately once a month but not weekly)
- Moderate / sometimes (about once a week)
- Severe / often (several times a week but not daily)
- Very severe / very often (daily)

Within these options, you will find information on both the severity and the frequency of the behavioural disorder since depending on the behavioural disorder, it can be better described with severity or frequency.

Please **answer all statements** and choose **only one** answer option per statement.

| **1** | **Does she/he show socially inappropriate behaviour, such as:** | **NO** | **Very mild /**  **Rare** | **Mild /**  **Occasio-nally** | **Moderate /**  **Some-times** | **Severe  /**  **Often** | **Very severe / Very often** |
| --- | --- | --- | --- | --- | --- | --- | --- |
| 1.1 | Inappropriate approaches, such as touching strangers or getting very close physically |  |  |  |  |  |  |
| 1.2 | Touching or kissing strangers |  |  |  |  |  |  |
| 1.3 | Verbal aggression, such as blaming, yelling at, etc. |  |  |  |  |  |  |
| 1.4 | Physical aggression, such as hitting, pushing, scratching, etc. |  |  |  |  |  |  |
| 1.5 | Urinating in public |  |  |  |  |  |  |
| 1.6 | Criminal behaviour (such as stealing) |  |  |  |  |  |  |
| Other examples or comments: | | | | | | | |
|  | | | | | | | |
|  | | | | | | | |

| **2** | **Have you noticed in her/him a loss of manners or etiquette, such as:** | **NO** | **Very mild /**  **Rare** | **Mild /**  **Occasio-nally** | **Moderate /**  **Some-times** | **Severe  /**  **Often** | **Very severe / Very often** |
| --- | --- | --- | --- | --- | --- | --- | --- |
| 2.1 | Inappropriate laughter |  |  |  |  |  |  |
| 2.2 | Swearing or yelling |  |  |  |  |  |  |
| 2.3 | Offensive comments |  |  |  |  |  |  |
| 2.4 | Rude or sexually suggestive comments |  |  |  |  |  |  |
| 2.5 | Lack of etiquette (e.g., not being able to wait in line) |  |  |  |  |  |  |
| 2.6 | Lack of respect |  |  |  |  |  |  |
| 2.7 | Failure to respond appropriately to social cues (e.g., continuing to talk even though it was signalled to stop) |  |  |  |  |  |  |
| 2.8 | Lack of personal hygiene (e.g., she/he wears malodorous, stained, torn or inappropriate clothing) |  |  |  |  |  |  |
| 2.9 | Rude behaviour in public, such as farting, scratching private parts, picking teeth, spitting, or belching |  |  |  |  |  |  |
| Other examples or comments: | | | | | | | |
|  | | | | | | | |
|  | | | | | | | |

| **3** | **Does she/he show impulsive, thoughtless, or careless actions, such as:** | **NO** | **Very mild /**  **Rare** | **Mild /**  **Occasio-nally** | **Moderate /**  **Some-times** | **Severe  /**  **Often** | **Very severe / Very often** |
| --- | --- | --- | --- | --- | --- | --- | --- |
| 3.1 | Reckless (car-)driving |  |  |  |  |  |  |
| 3.2 | Unwise buying or selling of products |  |  |  |  |  |  |
| 3.3 | Careless disclosure of personal data such as credit card number |  |  |  |  |  |  |
| Other examples or comments: | | | | | | | |
|  | | | | | | | |
|  | | | | | | | |

| **4** | **Have you noticed that she/he lacks drive, such as:** | **NO** | **Very mild /**  **Rare** | **Mild /**  **Occasio-nally** | **Moderate /**  **Some-times** | **Severe  /**  **Often** | **Very severe / Very often** |
| --- | --- | --- | --- | --- | --- | --- | --- |
| 4.1 | Lack of spontaneity |  |  |  |  |  |  |
| 4.2 | Decreased or lack of interest in activities that used to be important to her/him |  |  |  |  |  |  |
| 4.3 | Prompts are necessary for everyday tasks (such as brushing teeth) to be started or performed |  |  |  |  |  |  |
| 4.4 | Conversations are not initiated or maintained |  |  |  |  |  |  |
| Other examples or comments: | | | | | | | |
|  | | | | | | | |
|  | | | | | | | |

| **5** | **Have you noticed that he/she is less responsive to the needs and feelings of others, such as:** | **NO** | **Very mild /**  **Rare** | **Mild /**  **Occasio-nally** | **Moderate /**  **Some-times** | **Severe  /**  **Often** | **Very severe / Very often** |
| --- | --- | --- | --- | --- | --- | --- | --- |
| 5.1 | Ignorance of pain or suffering of others |  |  |  |  |  |  |
| 5.2 | Making hurtful comments leading to other person’s pain or suffering |  |  |  |  |  |  |
| Other examples or comments: | | | | | | | |
|  | | | | | | | |
|  | | | | | | | |
|  | | | | | | | |
| **6** | **Have you noticed in her/him a decreased interest in social contacts and relationships, such as:** | **NO** | **Very mild /**  **Rare** | **Mild /**  **Occasio-nally** | **Moderate /**  **Some-times** | **Severe  /**  **Often** | **Very severe / Very often** |
| 6.1 | Decreased interest in the company of others |  |  |  |  |  |  |
| 6.2 | Avoiding eye contact |  |  |  |  |  |  |
| 6.3 | Decrease in social engagement |  |  |  |  |  |  |
| Other examples or comments: | | | | | | | |
|  | | | | | | | |
|  | | | | | | | |

| **7** | **Have you noticed in her/him a decreased warmth in interpersonal interactions, such as:** | **NO** | **Very mild /**  **Rare** | **Mild /**  **Occasio-nally** | **Moderate /**  **Some-times** | **Severe  /**  **Often** | **Very severe / Very often** |
| --- | --- | --- | --- | --- | --- | --- | --- |
| 7.1 | Avoiding physical contact, such as touching or hugging friends and relatives |  |  |  |  |  |  |
| 7.2 | Emotional detachment, i.e., no longer reacting emotionally to external stimuli of a positive or negative nature |  |  |  |  |  |  |
| 7.3 | Emotional coldness |  |  |  |  |  |  |
| Other examples or comments: | | | | | | | |
|  | | | | | | | |
|  | | | | | | | |

| **8** | **Have you observed that she/he repeatedly performs the same movements, such as:** | **NO** | **Very mild /**  **Rare** | **Mild /**  **Occasio-nally** | **Moderate /**  **Some-times** | **Severe  /**  **Often** | **Very severe / Very often** |
| --- | --- | --- | --- | --- | --- | --- | --- |
| 8.1 | Rubbing hands |  |  |  |  |  |  |
| 8.2 | Tapping with hands or feet |  |  |  |  |  |  |
| 8.3 | Clapping hands |  |  |  |  |  |  |
| 8.4 | Scratching oneself |  |  |  |  |  |  |
| 8.5 | Tugging at skin or clothes |  |  |  |  |  |  |
| 8.6 | Humming |  |  |  |  |  |  |
| 8.7 | Clearing throat |  |  |  |  |  |  |
| 8.8 | Smacking of lips |  |  |  |  |  |  |
| Other examples or comments: | | | | | | | |
|  | | | | | | | |
|  | | | | | | | |

| **9** | **Have you noticed any compulsive or ritualistic behaviours in her/him, such as:** | **NO** | **Very mild /**  **Rare** | **Mild /**  **Occasio-nally** | **Moderate /**  **Some-times** | **Severe  /**  **Often** | **Very severe / Very often** |
| --- | --- | --- | --- | --- | --- | --- | --- |
| 9.1 | Compulsive counting |  |  |  |  |  |  |
| 9.2 | Compulsive cleaning rituals |  |  |  |  |  |  |
| 9.3 | Compulsive collecting or hoarding |  |  |  |  |  |  |
| 9.4 | Compulsive controlling |  |  |  |  |  |  |
| 9.5 | Compulsive going to the toilet |  |  |  |  |  |  |
| 9.6 | Compulsive arrangement of objects |  |  |  |  |  |  |
| 9.7 | Compulsive walking of certain routes |  |  |  |  |  |  |
| 9.8 | Compulsive repetition of words, phrases, or narratives |  |  |  |  |  |  |
| Other examples or comments: | | | | | | | |
|  | | | | | | | |
|  | | | | | | | |

| **10** | **Have you noticed any changes in her/his food preferences, such as:** | **NO** | **Very mild /**  **Rare** | **Mild /**  **Occasio-nally** | **Moderate /**  **Some-times** | **Severe  /**  **Often** | **Very severe / Very often** |
| --- | --- | --- | --- | --- | --- | --- | --- |
| 10.1 | Increased craving for sweets |  |  |  |  |  |  |
| 10.2 | Restriction on consumption of certain foods |  |  |  |  |  |  |
| Other examples or comments: | | | | | | | |
|  | | | | | | | |
|  | | | | | | | |

|  |  | **NO** | **Very mild /**  **Rare** | **Mild /**  **Occasio-nally** | **Moderate /**  **Some-times** | **Severe  /**  **Often** | **Very severe / Very often** |
| --- | --- | --- | --- | --- | --- | --- | --- |
| **11** | **Have you ever noticed her (him having any binge eating episodes?** |  |  |  |  |  |  |
| **12** | **Has she/he newly started consuming cigarettes or alcohol, or increased the usual consumption of cigarettes or alcohol?** |  |  |  |  |  |  |
| Other examples or comments: | | | | | | | |
|  | | | | | | | |
|  | | | | | | | |

| **13** **If you have noticed behavioural abnormalities in your close person that we have not asked about, please indicate them here:** |
| --- |
|  |
|  |
|  |
|  |
|  |
|  |

| **14 How frequently are you in touch with the close person? Please select one of the options below:** | |
| --- | --- |
|  | *Daily* |
|  | *Once or several times per week* |
|  | *Once or several times per month* |
|  | *Every other month* |
|  | *About once per year* |
|  | *Every other year* |

# Supplementary D. Kendall-Tau rank correlation coefficients

|  |  |  | **bvFTD vs. MDD** | **bvFTD vs. ADD** |
| --- | --- | --- | --- | --- |
|  |  |  | Kendell-Tau | Kendell-Tau |
| Domain_A | Subdomain A1 | Item_1_1 | .32 | .34 |
|  |  | Item _1_2 | .32 | .27 |
|  |  | Item _1_3 | ***.07^*^*** | ***.07^*^*** |
|  |  | Item _1_4 | .21 | .25 |
|  |  | Item _1_5 | .22 | .19 |
|  |  | Item _1_6 | .19 | .21 |
|  | Subdomain A2 | Item _2_1 | .50 | .50 |
|  |  | Item _2_2 | .18 | .17 |
|  |  | Item _2_3 | .36 | .39 |
|  |  | Item _2_4 | .28 | .21 |
|  |  | Item _2_5 | .55 | .47 |
|  |  | Item _2_6 | .40 | .38 |
|  |  | Item _2_7 | .39 | .43 |
|  |  | Item _2_8 | .48 | .43 |
|  |  | Item _2_9 | .49 | .43 |
|  | Subdomain A3 | Item _3_1 | ***.08^*^*** | ***.08^*^*** |
|  |  | Item _3_2 | .36 | .38 |
|  |  | Item _3_3 | .19 | .25 |
| Domain_B | Subdomain B1 | Item _4_1 | .19 | .37 |
|  |  | Item _4_2 | .27 | .45 |
|  | Subdomain B2 | Item _4_3 | .45 | .44 |
|  |  | Item _4_4 | .47 | .49 |
| Domain_C | Subdomain C1 | Item _5_1 | .54 | .57 |
|  |  | Item _5_2 | .31 | .26 |
|  | Subdomain C2 | Item _6_1 | .29 | .31 |
|  |  | Item _6_2 | .23 | .39 |
|  |  | Item _6_3 | .24 | .32 |
|  |  | Item _7_1 | .24 | .41 |
|  |  | Item _7_2 | .39 | .57 |
|  |  | Item _7_3 | .34 | .49 |
| Domain_D | Subdomain D1 | Item _8_1 | .25 | .37 |
|  |  | Item _8_2 | .23 | .33 |
|  |  | Item _8_3 | .25 | .23 |
|  |  | Item _8_4 | .25 | .30 |
|  |  | Item _8_5 | .16 | .25 |
|  |  | Item _8_6 | .24 | ***.06^*^*** |
|  |  | Item _8_7 | .27 | .12 |
|  |  | Item _8_8 | .27 | .31 |
|  | Subdomain D2 | Item _9_1 | .15 | .26 |
|  |  | Item _9_2 | ***.09^*^*** | .15 |
|  |  | Item _9_3 | .26 | .20 |
|  |  | Item _9_4 | ***-.03^*^*** | ***.05^*^*** |
|  |  | Item _9_5 | .21 | .36 |
|  |  | Item _9_6 | ***0^*^*** | ***.06^*^*** |
|  |  | Item _9_7 | .35 | .39 |
|  | Subdomain D3 | Item _9_8 | .30 | .24 |
| Domain_E | Subdomain E1 | Item _10_1 | .43 | .43 |
|  |  | Item _10_2 | .25 | .19 |
|  | Subdomain E2 | Item _11 | .26 | .49 |
|  |  | Item _12 | .21 | .27 |

^*^excluded items with *p*>.2

bvFTD = behavioural variant frontotemporal dementia, MDD = major depressive disorder, ADD = Alzheimer’s disease dementia, Domain A = Early behavioural disinhibition (Subdomain A1 = Socially inappropriate behaviour, Subdomain A2 = Loss of manners or decorum, Subdomain A3 = Impulsive, rash or careless actions); Domain B = Early apathy or inertia (Subdomain B1 = Apathy, Subdomain B2 = Inertia); Domain C = Early loss of sympathy or empathy (Subdomain C1 = Diminished response to other people’s needs and feelings, Subdomain C2 = Diminished social interest, interrelatedness or personal warmth); Domain D = Early perseverative, stereotyped or compulsive/ritualistic behaviour (Subdomain D1 = Simple repetitive movements, Subdomain D2 = Complex, compulsive or ritualistic behaviours, Subdomain D3 = Stereotypy of speech); Domain E = Hyperorality and dietary changes (Subdomain E1 = Altered food preferences, Subdomain E2 = Binge eating, increased consumption of alcohol or cigarettes). Categorisation of the domains and subdomains according to International Behavioural Variant FTD Criteria Consortium (FTDC) developed revised diagnostic criteria in 2011 [1]. Subdomain E3 was excluded from the Behavioural Dysfunction Questionnaire for lack of appearance in patients with mild-stage behavioural variant frontotemporal dementia [13].

# Supplementary E. Univariate logistic regression analyses with subdomains’ mean scores

|  | **bvFTD vs. MDD** | **bvFTD vs. ADD** |
| --- | --- | --- |
|  | **Regression coefficients [95% CI]** | **Regression coefficients [95% CI]** |
| Subdomain A1 | 3.4 [1.03-5.77] | 2.54 [1.15-3.93] |
| Subdomain A2 | 2.64 [1.62-3.66] | 2.79 [1.89-3.69] |
| Subdomain A3 | .99 [.4-1.58] | 1.07 [.55-1.59] |
| Subdomain B1 | .35 [.11-.59] | .84 [.57-1.11] |
| Subdomain B2 | .82 [.53-1.11] | 1 [.71-1.29] |
| Subdomain C1 | 1.26 [.77-1.75] | 1.19 [.78-1.6] |
| Subdomain C2 | .48 [.23-.73] | 1.04 [.69-1.39] |
| Subdomain D1 | 1.32 [.61-2.03] | 1.88 [1.11-2.65] |
| Subdomain D2 | 1.18 [.55-1.81] | 2.17 [1.31-3.03] |
| Subdomain D3 | .41 [.19-.63] | .33 [.15-.51] |
| Subdomain E1 | .82 [.47-1.17] | .79 [.5-1.08] |
| Subdomain E2 | .59 [.24-.94] | 1.34 [.85-1.83] |

CI = confidence interval

Please see Supplementary C for the names of the subdomains and the abbreviations.

# Supplementary F. Cross-validation for bvFTd vs. Mdd patients

| **Training models** | | | | | |
| --- | --- | --- | --- | --- | --- |
|  | **Round 1**  (n_bvFTD_=40, n_MDD_=57) | **Round 2**  (n_bvFTD_=40, n_MDD_=57) | **Round 3**  (n_bvFTD_=40, n_MDD_=57) | **Round 4**  (n_bvFTD_=40, n_MDD_=56) | **Round 5**  (n_bvFTD_=40, n_MDD_=56) |
|  | Regression coefficients [95% confidence interval] | | | | |
| Subdomain A1 | 3.2 [.77-5.63] | 2.77 [.57-4.97] | 3.27 [1.02-5.52] | 3.01 [.6-5.42] | 8.35 [.92-15.78] |
| Subdomain A2 | 3.04 [1.81-4.27] | 2.29 [1.23-3.35] | 2.53 [1.49-3.57] | 2.18 [1.22-3.14] | 3.71 [2.14-5.28] |
| Subdomain A3 | 1.34 [.56-2.12] | .85 [.2-1.5] | 1.17 [.46-1.88] | .86 [.25-1.47] | .82 [0.19-1.45] |
| Subdomain B1 | .4 [.13-.67] | .37 [.12-.62] | .39 [0.12-.66] | .22 [-.03-.47] | .39 [.12-.66] |
| Subdomain B2 | .79 [.46-1.12] | .92 [.57-1.27] | .88 [.53-1.23] | .79 [.46-1.12] | .75 [.44-1.06] |
| Subdomain C1 | 1.36 [.79-1.93] | 1.31 [.74-1.88] | 1.38 [0.81-1.95] | 1.18 [.63-1.73] | 1.12 [.61-1.63] |
| Subdomain C2 | .54 [.25-.83] | .52 [.25-.79] | .52 [0.23-.81] | .38 [.09-.67] | .45 [.18-.72] |
| Subdomain D1 | 1.08 [.37-1.79] | 1.13 [.35-1.91] | 2.28 [1.18-3.38] | 1.21 [.48-1.94] | 1.19 [.43-1.95] |
| Subdomain D2 | 1.2 [.47-1.93] | 1.34 [.6-2.08] | 1.12 [.49-1.75] | 1.16 [.45-1.87] | 1.15 [.44-1.86] |
| Subdomain D3 | .32 [.07-.57] | .4 [.16-.64] | .49 [.24-.74] | .41 [.17-.65] | .41 [.44-1.86] |
| Subdomain E1 | .82 [.43-1.21] | .89 [.48-1.3] | .85 [.46-1.24] | .87 [.48-1.26] | .68 [.33-1.03] |
| Subdomain E2 | .47 [.06-.88] | .6 [.19-1.01] | .59 [.2-.98] | .72 [.31-1.13] | .58 [.21-.95] |
|  | Regression coefficients | | | | |
| Domain A | 1.24 | .48 | .96 | .43 | .99 |
| Domain B | .06 | .24 | .15 | .2 | .13 |
| Domain C | .07 | . | . | . | . |
| Domain D | . | .2 | .86 | .06 | .12 |
| Domain E | . | .05 | .1 | .21 | .01 |
| **Test models** | | | | | |
|  | **Round 1**  (n_bvFTD_=10, n_MDD_=14) | **Round 2**  (n_bvFTD_=10, n_MDD_=14) | **Round 3**  (n_bvFTD_=10, n_MDD_=14) | **Round 4**  (n_bvFTD_=10, n_MDD_=14) | **Round 5**  (n_bvFTD_=10, n_MDD_=15) |
| AUC | 85.71% | 90% | 76.79% | 93.57% | 86.67% |
| Cut-off score | .21 | .36 | .19 | .21 | .1 |
| Sensitivity | 80% | 80% | 90% | 100% | 100% |
| Specificity | 86% | 100% | 79% | 93% | 67% |

Please see Supplementary C for the names of the domains and subdomains and the abbreviations.

# Supplementary G. Cross-validation for bvFTd vs. Add patients

| **Training models** | | | | | |
| --- | --- | --- | --- | --- | --- |
|  | **Round 1**  (n_bvFTD_=40, n_ADD_=96) | **Round 2**  (n_bvFTD_=40, n_ADD_=96) | **Round 3**  (n_bvFTD_=40, n_ADD_=96) | **Round 4**  (n_bvFTD_=40, n_ADD_=96) | **Round 5**  (n_bvFTD_=40, n_ADD_=96) |
|  | Regression coefficients [95% confidence interval] | | | | |
| Subdomain A1 | 3.97 [1.36-6.58] | 2.52 [.87-4.17] | 2.51 [1.14-3.88] | 2.16 [.79-3.53] | 2.3 [.87-3.73] |
| Subdomain A2 | 2.8 [1.82-3.78] | 2.46 [1.52-3.4] | 2.9 [1.9-3.9] | 2.95[1.87-4.03] | 2.91[1.85-3.97] |
| Subdomain A3 | 1.26 [.63-1.89] | .79[.26-1.32] | 1.22 [.61-1.83] | 1.27 [.6-1.94] | .91 [.36-1.46] |
| Subdomain B1 | .83 [.54-1.12] | .78 [.49-1.07] | .95 [.62-1.28] | .84 [.53-1.15] | .83 [.52-1.14] |
| Subdomain B2 | 1.02 [.69-1.35] | .91 [.6-1.22] | 1.09 [.74-1.44] | 1.05 [.7-1.4] | .99 [.66-1.32] |
| Subdomain C1 | 1.22 [.75-1.69] | 1.19 [.72-1.66] | 1.19 [.74-1.64] | 1.15 [.68-1.62] | 1.23 [.76-1.7] |
| Subdomain C2 | .96 [.61-1.31] | 1.12 [.71-1.53] | 1.14 [.73-1.55] | 1.02 [.61-1.43] | 1 [.63-1.37] |
| Subdomain D1 | 1.87 [1.01-2.73] | 1.54 [.76-2.32] | 1.97 [1.13-2.81] | 2.44 [1.36-3.52] | 1.75 [.93-2.57] |
| Subdomain D2 | 2.17 [1.15-3.19] | 2.06 [1.16-2.96] | 2.24 [1.3-3.18] | 2.27 [1.27-3.27] | 2.15 [1.19-3.11] |
| Subdomain D3 | .27 [.05-.49] | .38 [.18-.58] | .33 [.13-.53] | .39 [.17-.61] | .32 [.12-.52] |
| Subdomain E1 | .84 [.49-1.19] | .72 [.39-1.05] | .94 [.57-1.31] | .85 [.5-1.2] | .64 [.35-.93] |
| Subdomain E2 | 1.29 [.7-1.88] | 1.12 [.63-1.61] | 1.5 [.91-2.09] | 1.48 [.89-2.07] | 1.34 [.81-1.87] |
|  | Regression coefficients | | | | |
| Domain A | .85 | .47 | .59 | .63 | .61 |
| Domain B | .36 | .34 | .44 | .39 | .36 |
| Domain C | .01 | .22 | .11 | . | .11 |
| Domain D | .05 | .18 | .2 | .38 | .1 |
| Domain E | .06 | .12 | .45 | .24 | .13 |
| **Test models** | | | | | |
|  | **Round 1**  (n_bvFTD_=10, n_ADD_=24) | **Round 2**  (n_bvFTD_=10, n_ADD_=24) | **Round 3**  (n_bvFTD_=10, n_ADD_=24) | **Round 4**  (n_bvFTD_=10, n_ADD_=24) | **Round 5**  (n_bvFTD_=10, n_ADD_=24) |
| AUC | 92.92% | 95% | 81.67% | 91.67% | 95.42% |
| Cut-off score | .19 | .38 | .32 | .43 | .18 |
| Sensitivity | 90% | 90% | 70% | 100% | 100% |
| Specificity | 83% | 100% | 96% | 83% | 79% |

Please see Supplementary C for the names of the domains and subdomains and the abbreviations

**References:**

[1]. Rascovsky K, Hodges JR, Knopman D*, et al.* Sensitivity of revised diagnostic criteria for the behavioural variant of frontotemporal dementia. *Brain*. 2011 **134:** 2456-2477.

[2]. McKhann GM, Knopman DS, Chertkow H*, et al.* The diagnosis of dementia due to Alzheimer’s disease: Recommendations from the National Institute on Aging-Alzheimer’s Association workgroups on diagnostic guidelines for Alzheimer's disease. *Alzheimer's & dementia*. 2011 **7:** 263-269.

[3]. Spreen O, Strauss E. *A Compendium of Neuropsychological Tests, 2nd edn.* New York: Oxford University Press, 1991.

[4]. Stroop JR. Studies of interference in serial verbal reactions. *Journal of Experimental Psychology*. 1935 **18:** 643-662.

[5]. Zimmermann P, Fimm B. *Testbatterie zur Aufmerksamkeitsprüfung-Version 2.2*: Psytest, 2009.

[6]. Wechsler D. *Wechsler Memory Scale-Revised*. New York: Psychological Corporation, 1987.

[7]. Morris JC, Mohs RC, Rogers H, Fillenbaum G, Heyman A. Consortium to establish a registry for Alzheimer's disease (CERAD) clinical and neuropsychological assessment of Alzheimer's disease. *Psychopharmacol Bull*. 1988 **24:** 641-652.

[8]. Spreen O, Benton A. *Neurosensory Centre Comprehensive Examination for Aphasia*. Victoria: University of Victori, 1977.

[9]. Isaacs B, Kennie AT. The Set test as an aid to the detection of dementia in old people. *Br J Psychiatry*. 1973 **123:** 467-470.

[10]. Regard M, Strauss E, Knapp P. Children's production on verbal and non-verbal fluency tasks. *Percept Mot Skills*. 1982 **55:** 839-844.

[11]. Folstein MF, Folstein SE, McHugh PR. “Mini-mental state”: a practical method for grading the cognitive state of patients for the clinician. *Journal of Psychiatric Research*. 1975 **12:** 189-198.

[12]. Nasreddine ZS, Phillips NA, Bédirian V*, et al.* The Montreal Cognitive Assessment, MoCA: a brief screening tool for mild cognitive impairment. *Journal of the American Geriatrics Society*. 2005 **53:** 695-699.

[13]. Semenkova A, Piguet O, Johnen A*, et al.* The Behavioural Dysfunction Questionnaire discriminates behavioural variant frontotemporal dementia from Alzheimer’s disease dementia and major depressive disorder. *Journal of Neurology*. 2023 **270:** 3433-3441.
